# Effect of Butylphthalide combined with Oxiracetam on cognitive function, Intellectual recovery and serum inflammatory factors in patients with cognitive impairment after cerebral infarction

**DOI:** 10.12669/pjms.39.2.6901

**Published:** 2023

**Authors:** Wenxin Jiang, Xiao-dong Yu, Yanqing Deng

**Affiliations:** 1 Wenxin Jiang, Department of Neurology, Renmin Hospital, Hubei University of Medicine, Shiyan, Hubei 442000, P.R. China; 2 Xiao-dong Yu, Department of Neurology, Renmin Hospital, Hubei University of Medicine, Shiyan, Hubei 442000, P.R. China; 3 Yanqing Deng, Department of Neurology, Renmin Hospital, Hubei University of Medicine, Shiyan, Hubei 442000, P.R. China

**Keywords:** Butylphthalide, Oxiracetam, Cognitive impairment after infarction, Treatment

## Abstract

**Objective:**

To evaluate the effect of butylphthalide combined with oxiracetam on cognitive function, intellectual recovery and serum inflammatory factors in patients with cognitive impairment after cerebral infarction.

**Methods:**

This is a Clinical comparative study. A total of 80 patients with cognitive impairment after cerebral infarction who visited Renmin Hospital, Hubei University of Medicine from January 2020 to January 2022 were enrolled and randomly divided into two groups. Patients in the control group were treated with oral oxiracetam combined with routine treatment. Patients in the study group were given butylphthalide combined with oxiracetam on the basis of routine treatment. Compare the clinical effect, cognitive function and intellectual recovery, inflammatory factor level changes, CBV, CBF, MTT and other cerebral blood flow perfusion indicators, as well as post-treatment incidence of adverse drug reactions in the two groups of patients.

**Results:**

The efficacy of the study group was significantly higher than that of the control group (p=0.03). After treatment, the levels of CBV and CBF in the study group were higher than those in the control group, the levels of TNF-α, CRP and IL-6 were significantly lower than those of the control group, while MTT was shorter than that in the control group, with statistically significant difference (p=0.00). Furthermore, there was a statistically significant difference that the MMSE score and MOCA score of the study group were higher than those of the control group (p=0.00).

**Conclusion:**

Butylphthalide combined with oxiracetam has an obvious curative effect in the treatment of patients with cognitive impairment after cerebral infarction. It is a safe and effective therapeutic option that can significantly recover cognitive function and intelligence, improve cerebral blood flow perfusion and reduce inflammatory factors, without an obvious increase in adverse reactions.

## INTRODUCTION

Cerebral infarction refers to localized ischemic necrosis of brain tissue caused by local blood circulation disturbance in the brain.^1^ At present, symptomatic support is a common choice and has achieved a certain effect on the treatment of patients with cerebral infarction. However, it may induce potential neurological impairment in most patients, such as cognitive impairment, limb numbness, paralysis and other sequelae, and even the loss of independent living ability, which will bring a heavy burden to patients, their families and even society.^2^ Cognitive impairment is one of the common complications after cerebral infarction. Cannistraro et al.^3^ have reported that the incidence of cognitive impairment was 45% in patients with cerebral infarction, yet with unclear causes at present.^4^ Without timely and effective intervention, it can progress to vascular dementia, which will seriously affect the quality of life of the affected patients. According to related research^5^, improving the microcirculation of the brain and increasing the blood flow of the brain are of great significance for alleviating the degeneration and necrosis of nerve cells caused by hypoxia and ischemia. Butylphthalide is a multi-target drug for the treatment of cerebrovascular diseases, which can reduce the damage to the nervous system and the destruction of the blood-brain barrier by inhibiting the transformation of M1 microglia/macrophages to M2 phenotype.^6^

Moreover, it can also resume the living ability and neurological function of patients, and improve daily living activities by increasing the plasma level of 3-MST and decreasing the plasma level of Aβ42.^7^ Butylphthalide has been recognized to have a strong protective effect against cerebral ischemia. Meanwhile, oxiracetam is an emerging nootropic drug in recent years, which can improve memory impairment, repair damaged nerve cells, protect hypoxic brain tissue, and enhance learning ability.^8^ With respect to the above, this study was performed to discuss the clinical effect of butylphthalide combined with oxiracetam based on the grouping and comparative analysis of 80 patients with cognitive impairment treated in Renmin Hospital, Hubei University of Medicine.

## METHODS

This is a Clinical comparative study. A total of 80 patients with cognitive impairment after cerebral infarction who were admitted to Renmin Hospital, Hubei University of Medicine from January 2020 to January 2022 were enrolled in this study and randomly divided into two groups, with 40 cases in each group. There were 24 males and 16 females in the study group, with an average age of 63.50±6.58 (57~70) years old; and there were 22 males and 18 females in the control group, with an average age of 62.58±7.25 (55~70) years old. There was no significant difference in the general data between the two groups, suggesting the existence of comparability between groups (Table-I). The study was approved by the Institutional Ethics Committee of Renmin Hospital (No.: syrmy2022-047; Date: March 15, 2022), Hubei University of Medicine, and written informed consent was obtained from all participants.

**Table-I:** Comparison of general data between the study group and the control group (*x̅*±*s*) n=40.

Indicators	Study group	Control group	t/χ^2^	p
Age (years)	63.50±6.58	62.58±7.25	0.59	0.55
Male (n %)	24 (60%)	22 (55%)	0.20	0.65
Course of disease (d)	5.03±2.74	5.18±2.32	0.26	0.79
***Past medial history***				
Hypertension	17(42.5%)	15(37.5%)	0.21	0.65
Diabetes	15(37.5%)	12(30%)	0.50	0.48
Heart disease	8(20%)	13(32.5%)	1.61	0.20
***Bad habit***				
Smoking history	10 (25%)	12(30%)	0.25	0.62
Alcoholism	8 (20%)	7(17.5%)	0.08	0.76
High-fat diet	8(20%)	11(27.5%)	0.62	0.43
MMSE score	14.26±3.44	14.63±3.27	0.49	0.62
MoCA score	16.75±2.83	16.04±2.77	1.13	0.26

p>0.05.

### Inclusion criteria:

- Patients who met the diagnostic and therapeutic criteria of acute ischemic infarction^9^;
- Patients aged less than 70 years old;
- Patients with cognitive impairment according to the Simple Intelligence Scale (MMSE Score; 10~20 points) and Montreal Cognitive Assessment (MoCA; <26 points)^10^;
- Patients with onset time <14 d;
- Patients with a complete medical history and high compliance;
- Patients who were informed the study protocol and provided written consent form (by the patients or family members).

### Exclusion criteria:

- Patients with the co-existence of diseases or mental diseases that can lead to cognitive impairment;
- Patients who cannot cooperate to complete the test;
- Patients with severe heart, liver and kidney dysfunction;
- Patients with incomplete clinical data;
- Female patients in pregnancy or lactation;
- Patients with other brain diseases such as intracerebral hemorrhage, subarachnoid hemorrhage, brain injury, and transient cerebral ischemia.

Patients in the control group were treated with oral oxiracetam (0.8 g, twice a day) for 12 weeks continuously combined with routine treatment. Routine therapies included basic treatment such as blood pressure-lowering, glucose-lowering, blood lipid-lowering and anticoagulation to fully ensure the stability of plaque, improve brain edema, reasonably control and reduce intracranial pressure, and neuronutrition, etc. Patients in the study group were given butylphthalide combined with oxiracetam on the basis of routine treatment. The specific schemes were intravenous drip of butylphthalide (25 mg, >50 min, twice a day, with an interval of ≥six hours) for two weeks continuously, and oral oxiracetam (0.8 g, twice a day) for 12 weeks continuously.

Outcome measures: 1) Clinical effect observation: The patients were comprehensively analyzed by MoCA score based on symptom improvement before and after treatment.^11^ The clinical effect was classified into the following types: *Significantly effective:* with significant improvement in clinical symptoms and signs, and MoCA score >six points; *Effective:* with certain improvement in clinical symptoms and signs, and MoCA score of between four~six points; *Ineffective:* without improvement in clinical symptoms and signs, and MoCA score <four points. The response rate was calculated based on the sum of patients with significant effective and effective outcomes. 2) *Comparative analysis of blood perfusion indexes:* Brain CT was performed to measure the levels of cerebral blood flow perfusion indexes such as cerebral blood volume (CBV), cerebral blood flow (CBF) and mean transit time (MTT) before treatment and two weeks after treatment.

### Cognitive function and intellectual recovery:

The cognitive function, neurological function recovery and intellectual recovery of patients before and after treatment were respectively evaluated using MMSE (normal: 27-30 points; mild cognitive impairment: 21-27 points; moderate: 10-20 points; and severe: 0-9 points) and MoCA (normal: ≥26 points; showing deteriorated cognitive function with the decrease of scores) scales.

### Adverse reactions:

Corresponding adverse reactions included fever, gastrointestinal reactions, rash, etc. *Analysis of changes in inflammatory factors:* An amount of five ml peripheral venous blood was drawn from each patient in the morning before and after treatment. The levels of inflammatory factors such as tumor necrosis factor-α (TNF-α), C-reactive protein (CRP), and interleukin-6 (IL-6) were detected by enzyme-linked immunosorbent assay (ELISA). All patients were followed up for 6 months and were assessed for recurrence.

### Statistical analysis:

All data were statistically analyzed by SPSS 20.0 software. The measurement data were expressed in (Ax±s). The inter-group and intra-group data analyses used two independent samples t-test and paired t-test, respectively, and χ^2^ test was used for the comparison of rate. Statistical difference was exhibited when P<0.05.

## RESULTS

The comparative analysis of clinical effects between the two groups showed that the response rate of the study group was 82.5%, and that of the control group was 60%, which was higher in the former group than that of the latter group, and the difference was statistically significant (p=0.03). Table-II

**Table-II:** Comparison of clinical efficacy between the two groups (*x̅*±*s*) n=40.

Groups	Significant effective	Effective	Ineffective	Response rate
Study group	18	15	7	33 (82.5%)
Control group	11	13	16	24 (60%)
χ^2^				4.01
p				0.03

p<0.05.

There was no significant difference in CBV, CBF and MTT between the two groups before treatment (P>0.05). After treatment, the levels of CBV and CBF in the study group were higher than those in the control group, and the difference was statistically significant (P=0.00); while MTT was shorter in the former group than that in the latter group, with statistically significant difference (p=0.00).Table-III.

**Table-III:** Comparison of the recovery of blood perfusion indexes between the two groups before and after treatment (*x̅*±*s*) n=40.

Indicators		Study group	Control group	t	p
CBV (×10^-2^mL/g)	Before treatment	1.03±0.31	1.05±0.33	0.28	0.78
	After treatment*	1.42±0.27	1.17±0.25	4.30	0.00
CBF×10^-2^mL/ (g/min)	Before treatment	0.53±0.12	0.55±0.10	0.81	0.42
	After treatment*	0.97±0.21	0.61±0.13	9.22	0.00
MTT (S)	Before treatment	1.43±0.20	1.40±0.17	0.72	0.47
	After treatment*	1.04±0.11	1.26±0.18	5.96	0.00

p<0.05

No significant difference was observed in MMSE and MoCA scores between the study group and the control group before treatment (p>0.05). After treatment, the MMSE and MoCA scores of the study group were higher than those of the control group, with a statistically significant difference (p=0.00). Table-IV

**Table-IV:** Comparison of cognitive function and intellectual recovery between the two groups before and after treatment (*x̅*±*s*) n=40.

Groups	MMSE score		MoCA score	

	Before treatment	After treatment*	Before treatment	After treatment*
Study group	15.41±3.86	19.46±3.73	16.68±3.07	23.54±3.82
Control group	15.70±3.72	16.67±3.62	16.50±2.89	19.70±4.27
t	0.34	3.39	0.27	4.02
p	0.73	0.00	0.79	0.00

* p<0.05.

The incidence of adverse drug reactions was 32.5% and 27.5% in the study group and the control group, respectively. No significant difference was found in the incidence of adverse drug reactions between the study group and the control group (p=0.63) Table-V

**Table-V:** Comparison of the incidence of adverse drug reactions between the two groups (*x̅*±*s*) n=40.

Groups	Fever	Gastrointestinal reaction	Allergy	Rash	Granulocytopenia	Incidence rate
Study group	4	3	2	3	1	13 (32.5%)
Control group	2	2	3	2	2	11 (27.5%)
χ^2^						0.24
p						0.63

p>0.05.

There was no significant difference in TNF-α, CRP, IL-6 and other indicators between the study group and the control group before treatment (p>0.05); while those levels were obviously decreased in the study group than those in the control group after treatment, showing a statistically significant difference (p=0.00). Table-VI

**Table-VI:** Comparison of the changes in inflammatory factors between the two groups before and after treatment (*x̅*±*s*) n=40.

Indicators		Study group	Control group	t	p
TNF-α (ng/L)	Before treatment	28.40±7.28	27.51±7.08	0.63	0.57
	After treatment*	11.68±3.64	16.40±4.76	5.52	0.00
CRP (mg/L)	Before treatment	23.74±7.09	22.97±7.16	0.67	0.51
	After treatment*	13.54±3.47	15.13±4.51	4.63	0.00
IL-6 (ng/L)	Before treatment	11.01±2.38	11.28±2.51	0.36	0.68
	After treatment*	5.71±1.03	7.83±2.40	6.32	0.00

* p<0.05.

## DISCUSSION

Cerebral infarction is a common brain disease in neurology clinically that is common in middle-aged and elderly people, which is characterized by a high disability rate and mortality rate.^12^ The mortality of acute cerebral infarction has been reported to be 5% - 15%.^13^ Weaver et al.^14^ revealed in their research that there was a high risk of cognitive impairment (about 50%) even after effective treatment. Cognitive impairment has common manifestations such as memory impairment or other cognitive impairment to some extent, which seriously affects patients’ daily life. Hadanny et al.^15^ even believed that there was significantly higher mortality of cerebral infarction patients with cognitive impairment than that of patients without cognitive impairment. The pathogenesis of cognitive impairment is complicated, and it is generally considered to be related to the damage to hippocampal tissue related to memory and learning resulting from ischemia and hypoxia in the infarcted area of the brain. The possible pathogenesis includes structural injury of brain tissue, abnormalities of neurotransmitters such as acetylcholine, norepinephrine, dopamine, 5-hydroxytryptamine, as well as inflammatory reaction and the involvement of oxygen free radicals.^16^

Symptomatic treatment was a common choice for patients with cognitive impairment after cerebral infarction in the past, which improved the clinical symptoms of patients to a certain extent. However, it showed no obvious impact in promoting cognitive function and intellectual recovery of the affected patients. Comprehensive treatment may benefit patients considering that cognitive impairment after cerebral infarction is a multifactorial disease.^17^ The neurotransmitter acetylcholine has been accorded an important role in supporting learning and memory processes. Oxiracetam is an annular derivant of γ-aminobutyric acid annular derivate that can effectively increase the content of acetylcholine and improve cognitive and intellectual dysfunction induced by cerebral ischemia and hypoxia. Moreover, it can directly enter brain tissue through the blood-cerebrospinal fluid barrier and act on damaged brain tissue,^18^ which is effective to improve neurological function in patients with cerebral infarction.

Butylphthalide is a synthetic compound originally isolated from seeds of Apium graveolens.^19^ It can improve the dysfunction in the central nervous system and promote the recovery of neural function in patients with acute cerebral infarction. According to a prior study on animal pharmacodynamics,^20^ butylphthalide could block ischemic stroke-induced brain damage in multiple pathological links, have a strong anti-cerebral ischemia effect, significantly reduce the infarct area of local cerebral ischemia in rats, alleviate brain edema, improve brain energy metabolism as well as microcirculation and blood flow in cerebral ischemic regions, and inhibit neuronal apoptosis. Wang et al.^21^ believed that butylphthalide could alleviate edema by reducing brain cell damage resulting from the improvement of blood flow and energy metabolism in local brain tissue via inhibiting the release of glutamate, which may contribute to the improvement of cognitive function. In our study, the response rate of the study group (82.5%) was higher than that of the control group (60%), and the difference was statistically significant (p=0.03). Meanwhile, there was a statistically significant difference that both MMSE and MoCA scores were significantly elevated following combined therapy using butylphthalide in the study group than that in the control group (p=0.00).

There exist possible abnormalities in blood components in patients with cerebral infarction, such as the increase and aggregation of cellulose, platelets, fibrinogen and other components, which may accelerate thrombosis to induce the occlusion of the distal artery, resulting in insufficient CBF, reduced CBV, and slowed MTT.^22^ Significantly, butylphthalide has preventive effects against cerebral thrombosis and anti-platelet aggregation. In the present study, CBV and CBF (p=0.00) were significantly higher, while MTT (P=0.00) was much shorter in patients with combined therapy using butylphthalide than those in patients receiving routine treatment. These findings suggest that combined treatment can improve cerebral blood flow perfusion in patients with cognitive impairment after cerebral infarction. In terms of the potential causes, butylphthalide can block multiple pathological links of brain injury caused by cerebral infarction, reduce the infarct area, promote brain energy metabolism, and improve brain microcirculation; besides, it can also inhibit platelet aggregation, and effectively prevent thrombosis, hence improving cerebral blood flow perfusion eventually.^23^

Furthermore, inflammatory reaction plays an important role in the pathological process of cognitive impairment after cerebral infarction.^24^ Cerebral ischemia can lead to the activation of endothelial cells, which can accelerate the release of IL-6, TNF-α and other inflammatory factors.^25^ Kamnaksh et al.^26^ have documented in their research that increased expression levels of IL-6 and TNF-α in the cerebrospinal fluid would indicate higher severity of the condition in patients with vascular cognitive impairment. Simultaneously, hippocampal gray matter volume is an important index to determine the severity of vascular cognitive impairment. The level is negatively correlated with the volume of hippocampal gray matter. Critically, butylphthalide may produce the aforementioned pharmacological effects by reducing the content of arachidonic acid, increasing the levels of nitric oxide (NO) and prostaglandin I2 (PGI2) in cerebral vascular endothelium, inhibiting the release of glutamate, decreasing intracellular calcium concentration, suppressing oxygen free radicals, improving antioxidant activity, etc.^27^ In view of the results of our study, there was no statistically significant difference in the levels of TNF-α, CRP and IL-6 between the study group and the control group before treatment (p>0.05); while the levels of those indicators decreased evidently following combined use of butylphthalide than those in the control group, with statistically significant difference (p=0.00).

### Limitations of this study:

It includes the small sample size, the lack of follow-up, and the absence of exploring the specific effect of butylphthalide combined with other treatment modalities on patients with cognitive impairment after cerebral infarction. In future clinical research, it is planned to evaluate the advantages and disadvantages of this treatment plan more comprehensively and objectively based on the expansion of sample size, the addition of follow-up data, etc., so as to benefit more patients clinically.

## CONCLUSION

Combined therapy using butylphthalide and oxiracetam can be regarded as a safe and effective therapeutic option in the treatment of patients with cognitive impairment after cerebral infarction. It can improve the cognitive function of the affected patients, which may be related to the improvement of cerebral blood flow perfusion and regulation of serum inflammatory factors, without an obvious increase in adverse reactions.

### Authors’ Contributions:

**WJ, XY** **and** **YD** designed this study, prepared this manuscript, are responsible and accountable for the accuracy and integrity of the work.

**WJ and** **XY** collected and analyzed clinical data.

**YD** Data analysis, significantly revised this manuscript.
